# Cellular Mechanisms of FGF-Stimulated Tissue Repair

**DOI:** 10.3390/cells10071830

**Published:** 2021-07-20

**Authors:** Igor Prudovsky

**Affiliations:** Maine Medical Center Research Institute, 81 Research Dr., Scarborough, ME 04074, USA; prudoi@mmc.org

**Keywords:** FGF, repair, regeneration, proliferation, stemness, de-differentiation, angiogenesis, cell death, inflammation, cell senescence, protease production, FGF secretion

## Abstract

Growth factors belonging to the FGF family play important roles in tissue and organ repair after trauma. In this review, I discuss the regulation by FGFs of the aspects of cellular behavior important for reparative processes. In particular, I focus on the FGF-dependent regulation of cell proliferation, cell stemness, de-differentiation, inflammation, angiogenesis, cell senescence, cell death, and the production of proteases. In addition, I review the available literature on the enhancement of FGF expression and secretion in damaged tissues resulting in the increased FGF supply required for tissue repair.

## 1. Introduction

The fibroblast growth factor (FGF) family comprises signaling proteins that perform a wide variety of biological functions, including the positive regulation of tissue and organ repair and regeneration. In this article, after briefly reviewing FGF signaling and summarizing the existing data on the stimulation of repair and regeneration by FGFs, I will focus on the cellular effects of FGFs, which underlie their pro-reparative activity. I will also discuss known mechanisms responsible for the increased supply of FGFs in damaged tissues.

## 2. FGF Family and FGF Signaling

**Figure 1:**
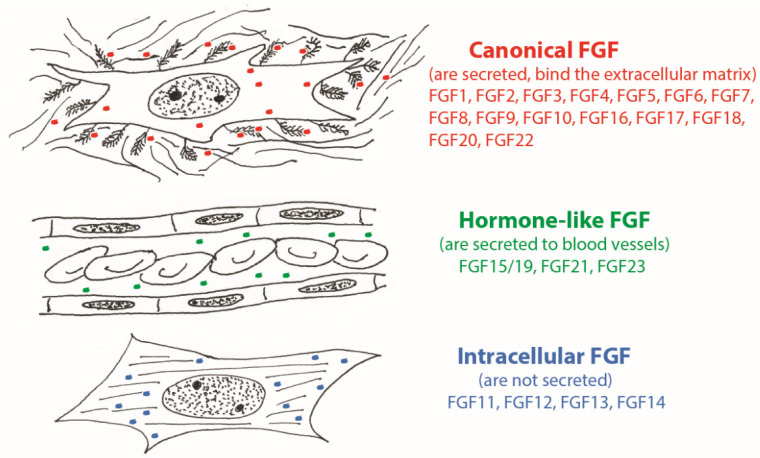
Three major groups of FGFs: canonical, hormone-like, and intracellular.

In mammals, there are 22 members of the FGF family (Figure 1). These are relatively small proteins (around 20 kDa), which have in their core a typical β-barrel structure [1]. Most FGFs (canonical FGFs) are secreted and function either as paracrine or autocrine growth factors. However, a subfamily of FGFs includes four intracellular proteins (FGF11-14) involved in the regulation of ion channels [2]. Another group (FGF 15/19, 21 and 23) consists of secreted hormone-like proteins regulating various aspects of organism metabolism [3].

Secreted FGFs signal through specific transmembrane protein kinase receptors (FGFR). There are four FGFR, and FGFR 1, 2, and 3 are presented by alternative splice variants (b or c) [4,5]. Hormone-like FGF15/19 (i.e., mouse FGF15 and its human ortholog FGF19), FGF21, and FGF23 require, for their activity, FGFR and the co-receptor transmembrane protein, Klotho [6]. Secreted canonical FGFs have a strong affinity for heparan sulfate proteoglycans (HSPG), which increase their association with the extracellular matrix (ECM) and underlie the local character of their activity. In contrast, hormone-like FGFs have a reduced HSPG affinity. HSPGs can protect secreted canonical FGFs from extracellular proteases [7], which are especially abundant in damaged tissues. Similar to their ligands, FGFRs bind HSPGs, resulting in the formation of FGF-HSPG-FGFR ternary complexes, which are required for the initiation of FGF signaling. The presence of FGFRs in most cell types and the expression of FGFs in all organs make them ubiquitous components of the locally available “first response kit”, ready to stimulate the repair of damaged tissues and organ regeneration.

**Figure 2:**
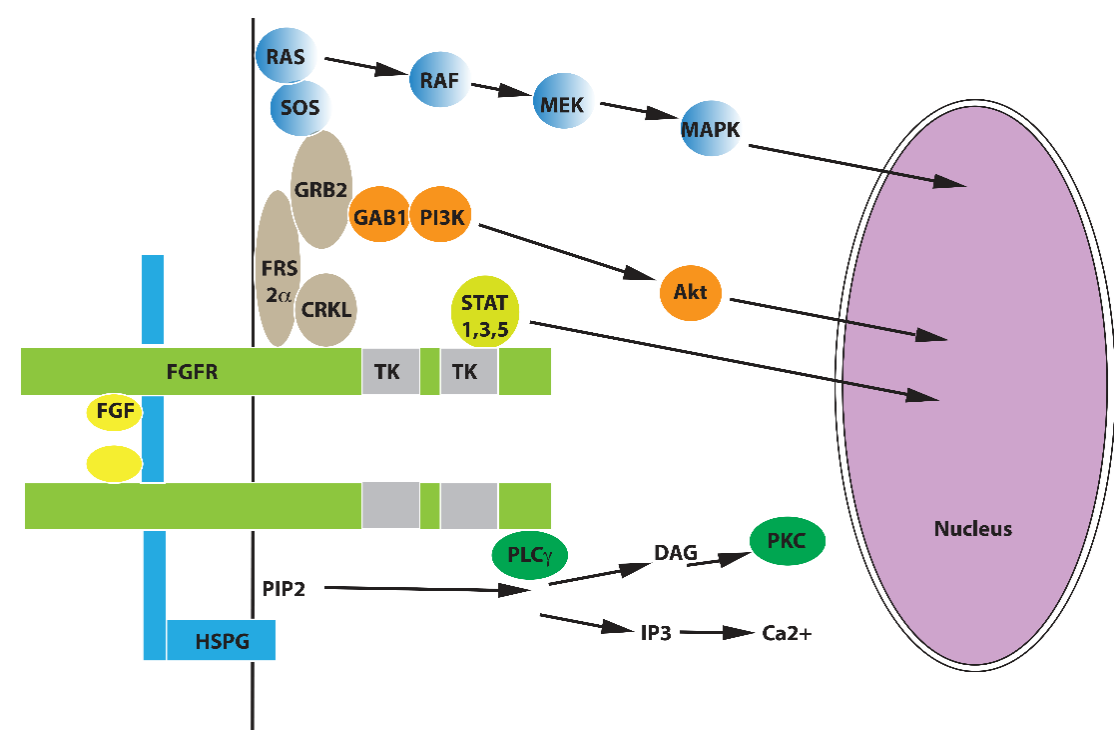
FGFR signaling pathways.

The formation of ternary complexes comprised of FGF, FGFR, and HSPG (Figure 2) results in the FGFR dimerization and phosphorylation of multiple tyrosine residues in the intracellular domain of FGFR, which is mediated by two tyrosine kinase domains of FGFR [4,5]. This phosphorylation enables the activation of several signaling pathways: (i) The binding of phospholipase C gamma (PLCγ) to one of FGFR phosphotyrosines leads to the activation of PLCγ. PLCγ degrades phosphatidylinositol 4,6-bisphosphate (PIP2) to inositol 1,4,5-triphospate (I3P), the inducer of calcium ions release from the endoplasmic reticulum, and to diacyl glycerol (DAG), the activator of protein kinase C (PKC); (ii) The binding of transcription factors STAT1, 3 and 5 to another phosphotyrosine of FGFR enables their phosphorylation and subsequent nuclear translocation; (iii) The binding of the adaptor protein, CRKL, to a specific phosphotyrosine of the FGFR results in the binding of CRKL to the major FGFR substrate, FRS2α, which is constitutively associated with the juxtamembrane part of the intracellular domain of FGFR. This facilitates the phosphorylation of FRS2α by FGFR, resulting in the consecutive recruitment of the adaptor protein, GRB2, and the guanine nucleotide exchange factor, SOS, which activates the Ras-MAPK signaling pathway; (iv) The activated FRS2 also recruits, through GRB2, the adaptor protein GAB1, which activates the PI3K-Akt signaling pathway. Moreover, the FGFR signaling is tightly controlled by a group of negative regulators, including SPRY1-4, SEF, DUSP6, SHP2, and CBL [5,8].

## 3. FGF as Stimulators of Regeneration and Repair

### 3.1. FGF and Regeneration in Invertebrates

FGFs appeared very early in the course of animal evolution—already in Cnidarians, the most primitive metazoans. Indeed, *Hydra* has four predicted genes coding for FGFs [9]. Unlike vertebrates, organ regeneration in most invertebrate phylae is a common event, especially in Cnidarians. Interestingly, a specific chemical inhibitor of FGFR suppressed head regeneration in *Hydra vulgaris* [10]. An FGF homolog has been discovered in *Planaria* flatworms [11]. At the early stage of planarian regeneration, following injury, an increased FGF expression was reported in the cells bordering the wound. An especially strong induction of the planarian FGF expression was detected in the course of head regeneration [11]. A heparin-binding polypeptide, with a structure similar to mammalian FGF2 and the ability to stimulate mammalian cell proliferation, was also found in the regenerating tissues of an echinoderm (i.e., sea star *Asterias rubens*) [12]. In the brittle sea star, *Amphiura filiformis,* FGF signaling was shown to be critically important for skeleton formation in regenerating arms [13].

### 3.2. FGF and Regeneration in Lower Vertebrates

Unlike higher vertebrates (birds and mammals), lower vertebrates, such as fishes and amphibians, exhibit a pronounced capacity to regenerate amputated organs. Zebrafish (*Danio rerio*) and the urodele amphibian axolotl (*Ambystoma mexicanum*) represent two excellent models to study organ regeneration, particularly the role of FGFs in this process. In 2000, Poss and colleagues demonstrated that a specific FGFR inhibitor suppressed the caudal fin regeneration in zebrafish [14]. This effect was mediated by the suppression of the formation of blastema, a mass of actively proliferating undifferentiated cells at the site of the fin amputation. A similar effect can be achieved by the overexpression of a dominant negative mutant of FGFR1 [15]. Keating et al. [16] found that FGF20 is essential for zebrafish fin regeneration. In zebrafish, FGF signaling was also shown to be required for the regeneration of the spinal cord [17], liver [18], heart [19], lateral line neuromast hair cells [20], rod photoreceptor cells [21], and extraocular muscle [22]. An enhanced expression of FGF8 was detected in regenerating larval limbs of the African frog, *Xenopus* [23], and axolotl [24], and an FGF inhibitor suppressed the regeneration of the *Xenopus* tadpole tail [25]. The cooperative application of FGF2, FGF8, and BMP7 to skin wounds in axolotls and newts resulted in the ectopic formation of limbs, instead of simple wound healing [26]. Limb regeneration in axolotl is known to be nerve-dependent [27]. It has been shown that FGF8 produced in the spinal ganglia of axolotl is delivered through long axons to regenerate limbs [28]. Collectively, the studies on invertebrate and lower vertebrate models demonstrate the importance of FGFs for organ regeneration.

### 3.3. FGF and Tissue Repair in Higher Vertebrates

Unlike invertebrates and lower vertebrates, higher vertebrates are almost completely devoid of the ability to regenerate organs, except for a few specific cases, including the re-growth of amputated digits in mice during the early post-natal development [29] and the regeneration of the liver after partial resection [30]. However, higher vertebrates efficiently repair tissues after mechanical wounding, burns, or chemical damage [31], and FGF signaling plays important roles in these repair processes [32]. For example, the total knockout of FGF2 [33] or simultaneous knockouts of FGFR1 and FGFR2 in keratinocytes [34] strongly delayed the healing of skin wounds in mice. The double knockout of FGFR1 and FGFR2 in oligodendrocytes impeded the repair of demyelinated lesions in a murine spinal cord [35]. FGFR2 knockout in mouse urothelium resulted in the suppression of urothelial regeneration after cyclophosphamide-induced damage [36]. Mice with total FGF2 knockout exhibited a poor epithelial recovery in the lungs after bleomycin-induced damage [37]. Interestingly, further studies using mice with an inducible FGF2 expression demonstrated that FGF2 also suppressed the bleomycin-induced lung fibrosis [38]. For a detailed review of the studies focused on the roles of FGFs and FGFRs in repair and regeneration in vertebrates, I recommend the excellent article by Sabine Werner and colleagues [32].

### 3.4. Use of Recombinant FGFs for Tissue Repair

Recombinant FGFs have been shown to stimulate the repair of various tissues in animal models, mostly mice and rats. Thus, application of FGF1 or FGF2 accelerated the closing of skin wounds [39], healing of diabetic ulcers [40], repair of damaged spinal cord [41], and healing of bone fractures [42]. In these studies, either recombinant FGF proteins or genetic constructs coding for them were applied. The efficiency of healing was improved using FGF mutants with a higher activity and increased stability [43] and also by the delivery of FGFs from slow-releasing gels [44]. The clinical use of FGFs for wound healing has been approved in China [45]. Several recent detailed reviews [45,46,47] focused on the application of recombinant FGFs for tissue repair are available.

### 3.5. Potential Participation of Intracellular and Hormone-Like FGFs in Repair Processes

While the participation of canonical (secreted HSPG-binding) FGFs in repair is well documented, the roles of intracellular FGF11-14 and hormone-like FGF15/19, 21 and 23 in this process remain insufficiently studied. Some published results indicate that intracellular FGFs may have the potential to stimulate tissue repair [48]. For example, FGF11, a factor that is induced under hypoxic conditions, stimulated in vitro angiogenesis and enhanced the bone-resorbing activity of osteoclasts [49]. Moreover, hypoxia-induced FGF11 interacts with HIF1α, the major transcription factor responsible for hypoxia response, and increases HIF1α stability [50]. The stimulation of tissue repair by hormone-like FGFs is also supported by a number of studies. For example, a knockout of their co-receptor, Klotho, resulted in delayed skin wound healing in mice and was accompanied by an increased expression of proinflammatory cytokines in wound lesions [51]. Moreover, the systemic administration of FGF21 attenuated neurodegeneration and neuroinflammation in aged and diabetic mice [52] and improved the recovery of spinal cord injury in rats [53]. Finally, *Fgf15*^−/−^ mice demonstrated a strongly suppressed ability to regenerate the liver after partial resection [54].

## 4. Cell Processes Underlying the Stimulation of Tissue Repair by FGF

**Figure 3:**
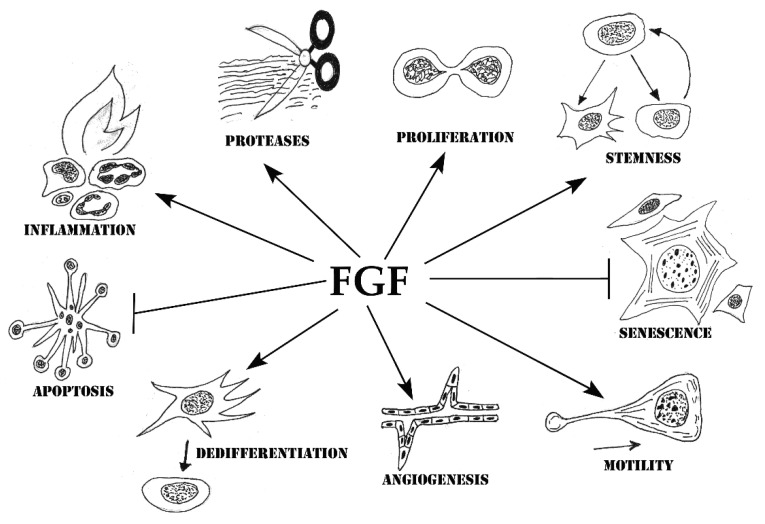
Cellular processes regulated by FGFs.

As demonstrated in a large number of in vitro and in vivo studies, FGFs regulate numerous aspects of the cell phenotype critical for successful tissue repair (Figure 3, Table 1).

### 4.1. Maintenance of Cell Stemness

Tissue repair depends on the activation of locally present stem and progenitor cells, resulting in their proliferation and subsequent differentiation to tissue-specific cell types. A number of in vitro studies have demonstrated the importance of FGFs for the maintenance of cell stemness, i.e., the ability of stem cells to maintain the non-differentiated status and to give rise to differentiated cells under proper conditions. Thus, FGF2 efficiently maintained the stemness of rabbit [90] and human [91] embryonic stem cells in culture, as well as the stemness of neural stem cells [92], trophoblast stem cells [93], and periodontal ligament stem cells [94] in mice. In addition, FGF2 and FGF4 supported the stemness of bone marrow-derived mesenchymal stem cells in vitro [95]. In vivo, FGF2 and FGF6 maintain the stemness of skeletal muscle satellite cells (for review see [96]). FGF signaling also maintained nephron progenitor cells [97] and preserved the stemness of prostate stem cells [98] in vivo. FGF8, by signaling through FGFR1, supported the undifferentiated status of spermatogonial stem cells [99]. In stem cells, FGF signaling was shown to maintain the expression of transcription factors SOX2 [100], OCT4 [101], and NANOG [102], which are key positive regulators of stemness.

### 4.2. Induction of Cell De-Differentiation

Tissue repair is accompanied by the partial de-differentiation of differentiated cells and their enhanced proliferation and migration, followed by the eventual return to the differentiated state [103]. FGFs have long been known to efficiently induce cell de-differentiation. For example, FGF3 treatment reversibly suppressed the differentiation characteristics of thyroid epithelial cell in vitro [75]. A similar effect of FGF2 was observed in a chondrocyte culture [81]. More recently Kleiderman et al. [104] have shown that the addition of recombinant FGF2 to a non-proliferating culture of stem cell-derived murine astrocytes stimulated their proliferation and conversion to neurogenic stem cells. Recent studies by Murota and colleagues [77] have demonstrated that in a skin wound treated with recombinant FGF2, the wound edge keratinocytes underwent an enhanced epithelial-mesenchymal transition (EMT), a de-differentiation process accompanied by the increased expression of EMT transcription factors, such as Snail 2, a decreased expression of the epithelial marker, E-cadherin, and the induced expression of the mesenchymal marker, vimentin. As a result, the wound healing was accelerated. Saera-Vila et al. [22] have shown that FGF signaling is required for the myocyte de-differentiation involved in the regeneration of the extraocular muscle of zebrafish. FGF-induced cell de-differentiation could also take place in pathological situations. Thus, while in vitro FGF induced the de-differentiation of vascular smooth muscle cells (VSMC) from a contractile to synthetic phenotype, the study of atherosclerotic plaques has shown an enhanced FGFR signaling and decreased expression of contractile proteins in VSMC [70]. The study by Chen et al. [71] revealed an antagonistic relation between the FGF and TGFβ signaling pathways in the regulation of the VSMC phenotype. The induction of FGF signaling inhibited TGFβ signaling and resulted in the synthetic phenotype of VSMC, while the inhibition of FGF signaling led to the enhancement of TGFβ signaling and the contractile phenotype. Moreover, the SMC-specific deletion of *Frs2α* strongly reduced the neointima formation after carotid ligation. It is noteworthy that while SMC show phenotypic similarities with myofibroblasts, FGF2 suppresses the differentiation of fibroblasts to myofibroblasts [72].

### 4.3. Proliferative Stimulation and Its Limitation

HSPG-binding FGFs stimulate the proliferation and migration of a wide variety of cell types, both in vitro and in vivo [5]. These effects depend on the presence of appropriate FGFRs at the target cell surface. The most versatile of FGFs is FGF1, which binds all known types of FGFRs [105]. The application of recombinant FGFs to repair various damaged tissues leads to a strongly enhanced cell proliferation [45,46,47].

In vitro, the application of FGFs to quiescent cells prompts their entry to the cell cycle, followed by DNA synthesis and mitosis [106]. We found that while at least some malignant cells can continuously proliferate in a serum-free medium supplemented with FGF1, in the cultures of non-transformed cells, such as Swiss 3T3 fibroblasts, the continuous stimulation with FGF1 in the serum-free medium leads to one round of DNA synthesis and mitosis, but it does not result in the second round of DNA synthesis [107], and the cells remain blocked in the second G1 phase of the cell cycle, relative to the onset of FGF-stimulation. Characteristically, these non-transformed cells fail to express the cyclin A2 needed for the initiation of DNA synthesis [107]. Interestingly, although the removal of FGF1 after the first FGF1-stimulated cell cycle results in the return to quiescence, re-stimulation with FGF1 still does not lead to the initiation of DNA synthesis. We named this phenomenon FGF memory. That is, cells “remember” the original FGF stimulation and remain proliferatively refractory to the repeated FGF stimulation for a period of at least one week. Furthermore, transient stimulation also with FGF2 [107] and FGF9 (unpublished) resulted in the establishment of FGF memory. The phenomenon of FGF memory was found not only in fibroblasts, but also in endothelial cells, as well as in bone-marrow-derived mesenchymal stem cells and adipose-derived stem cells [107]. Unlike FGFs, transient stimulation with PDGF-BB did not result in “PDGF memory” formation [107]. While IGF1, when applied as a single growth factor, fails to induce DNA synthesis in quiescent cells, its application, together with FGF1, to cells with a previous history of FGF stimulation nullified FGF memory, achieving a robust stimulation of DNA synthesis [107]. In this connection, it should be noted that the growth factor combinations, including both FGF and IGF, are more efficient stimulators of animal tissue repair than FGF alone [108,109]. Interestingly, cells arrested in the second G1 period during the continuous application of FGF still exhibited a strong migration [107]. Indeed, we have previously shown that the stimulation of migration and proliferation by FGF1 proceeds through different signaling pathways [110]. FGF memory can be eliminated by the application of the inhibitors of histone deacetylases, which indicates the epigenetic nature of this phenomenon [107]. One may suggest that the inefficiency or low efficiency of recombinant FGFs reported in some tissue repair studies could be due to the insufficient proliferation caused by the rapid establishment of FGF memory because of an insufficient local expression of additional growth factors, such as IGFs. We hypothesize that FGF memory is required for the regulation of cell growth in the process of tissue repair to prevent excessive cell proliferation and angiogenesis. It could also support vascular integrity by enabling the FGF-dependent maintenance of the viability and adhesion of endothelial cell, while preventing their growth.

### 4.4. Suppression of Cell Senescence

The aging of an organism is accompanied by the accumulation in various organs of senescent cells that have irreversibly lost the ability to proliferate [111]. A similar process occurs during the serial passaging of non-transformed cells in culture [112]. We have shown in vitro that both proliferative and migratory responses to FGF are impaired in senescent human endothelial cells [113]. Senescent cells exhibit a number of specific characteristics, including high levels of lysosomal beta-galactosidase and inhibitors of cyclin-dependent kinases, such as p16/Ink and p21/Cip, and the suppression of telomerase activity, resulting in a critical loss of telomere DNA [114]. The age-related decrease of tissue repair efficiency could at least partially be explained by the increased presence of the senescent cells, which are not only refractory to proliferation stimuli, but also secrete a variety of cytokines (e.g., interleukin 1α) that negatively regulate the proliferation of adjacent cells [115,116]. A number of studies have demonstrated that FGFs can delay cell senescence and extend the lifespan of cells, if applied before the cells acquire the senescent phenotype. For example, unlike VEGF-A, FGF2 strongly extended the proliferative lifespan of human endothelial cells in vitro and increased the activity of telomerase in these cells [55]. FGF2 also suppressed the senescence of human mesenchymal stem cells by drastically decreasing the expression of cell proliferation inhibitors, p16, p21, and p53 [88]. Fetal fibroblast cell lines that typically demonstrate longer lifespans than adult fibroblasts also exhibit a higher expression of FGF1 and FGF2 [117]. It has also been found that FGF2 upregulated the gene expression of *TERT* (telomerase reverse transcriptase) in human embryonic stem cells [118]. The medium conditioned by mouse embryonic stem cells suppressed the senescent phenotype of human fibroblasts by increasing the expression of FGF2 in fibroblasts [119]. This conditioned medium also accelerated wound healing in vivo [119]. Page et al. have shown that the addition of FGF2 to the culture medium increased the in vitro lifespan of human fibroblasts and stimulated the expression of stemness transcription factors OCT4, SOX2, and NANOG [73].

### 4.5. Suppression of Cell Death

Massive cell death is a typical result of traumatic injury. Suppressing cell death could limit the extent of tissue damage and promote a more efficient wound healing. FGFs are well documented to suppress apoptosis, a major mechanism of cell death. In vitro FGF2 decreased the apoptosis of oligodendrocytes [86], endothelial cells in corneas stored at 24 to 34 °C [56], and bone marrow-derived mesenchymal stem cells exposed to hypoxic conditions [87]. A topical injection of FGF7 (keratinocyte growth factor) protected the cells of hair follicles from apoptosis induced by UV irradiation [120]. FGF4 protected male germ cells in vitro from heat shock-induced apoptosis [89]. The knockdown of FGF9 in gastric cancer cell lines induced apoptosis, while the high expression of FGF9 in gastric cancers was correlated with a poor prognosis [121]. The inhibition of FGF signaling in glioma cells induced by a dominant negative FGFR mutant resulted in the activation of pro-apoptotic caspases 3 and 9 [122]. Russel et al. [84] found that the transgenic overexpression of FGF1 in rat brain protected neurons from apoptosis induced by perinatal hypoxia-ischemia and attenuated the activation of caspases 3 and 9. In addition to apoptosis, FGF treatment has been shown to suppress another cell death pathway, necroptosis. For example, FGF2 significantly decreased the peroxide-induced necrotic death of H9c2 cardiomyocytes [79]. The in vitro application of FGF2 to cardiomyocytes protected them from the toxic effect of doxorubicin [80], an anti-tumor chemotherapy agent, which affects myocardium. Due to the presence of alternative in-frame translation initiation codons in *FGF2* mRNA, FGF2 can be expressed as low (Lo-FGF2, 18 kDa) and high (Hi-FGF2, greater than 20 kDa) molecular weight isoforms, of which Hi-FGF2 forms, but not Lo-FGF2, exhibit nuclear localization [123]. Recently, Kardami et al. showed that while endogenous Lo-FGF2 produced by non-myocyte cardiac cells reduced the cardiotoxic effect of doxorubicin, on the contrary, endogenous Hi-FGF2 exacerbated it [124].

The molecular mechanisms underlying the anti-apoptotic effects of FGFs have been extensively studied. For example, Peluso [125] reported that the prevention of apoptosis in the culture of ovarian granulosa cells was due to the maintenance of a normal intracellular Ca^2+^ concentration achieved through the stimulation of calcium efflux by the plasma membrane calcium, ATPase (PMCA) [125]. The PMCA activation was dependent on its membrane localization stimulated by the protein kinase, Cδ, an enzyme activated by FGF signaling [125]. Kim et al. [126] have shown that the anti-apoptotic effect of FGF2, which prevents the death of ATDC5 cells treated with TNFα, was dependent on the induction of anti-apoptotic proteins, Bcl2-A1 and Bcl-xL. A recent study by Okada et al. [85], who used specific siRNAs and chemical inhibitors in vivo, demonstrated that the suppression by recombinant FGF2 of rat neuron death induced by subarachnoid hemorrhage was mediated by the FGFR3/PI3K/Akt signaling axis. Similar results were obtained by Tahara et al. [127], who found that the survival of zebrafish cardiomyocytes after heart injury was dependent on endogenous FGF-Akt signaling. The protective effect of FGF2 against the toxicity of doxorubicin in a cardiomyocyte culture was mediated through the pathway involving the mTOR signaling complex, Nrf-2 transcription factor, and the stress-induced protein, HO-1 [80].

### 4.6. Regulation of Inflammation

Trauma-induced inflammation is an important component of reparative processes, in which resident and invading inflammatory cells participate in tissue regeneration [128]. FGFs are well known as potent regulators of inflammation. Thus, Qi and Xin have reported that FGF2 induced proinflammatory cytokine expression in human aortic VSMCs and their transition from the contractile to the secretory phenotype [70]. The cytokine-induced activation of the pro-inflammatory NFκB signaling in hepatic stellate cells was shown to depend on the kinase activity of FGFR1 [129]. FGFR1 was also critically important for enhanced NFκB signaling in prostate cancer cells, and this effect of FGFR1 was dependent on the stabilization of the TAK1 kinase [130]. FGF7 induced the TNFα expression in immortalized human keratinocytes through the FGFR2/Akt/NFκB signaling axis [78]. In human articular chondrocytes, FGF2 stimulated the IL1β-dependent expression of the proinflammatory protein substance P and its receptor, NK_1_-R [82]. In this connection, it is noteworthy that NFκB signaling and IL1α expression are prerequisites of the aforementioned FGF memory [107].

FGF2 stimulates the infiltration of tissues by inflammatory cells, such as T lymphocytes [131], and macrophages [132]. We found that the transgenic overexpression of FGF1 in endothelial cells resulted in an exaggerated macrophage infiltration after kidney ischemia-reperfusion [133]. Similarly, Meij et al. [134] reported that the transgenic overexpression of FGF2 in cardiomyocytes enhanced the T lymphocyte infiltration into the heart after isoproterenol treatment.

In contrast to the studies demonstrating the NFκB-mediated proinflammatory effects of FGFs, several groups have reported the anti-inflammatory effects of FGF1 in vivo. For example, the herpes virus-mediated overexpression of FGF2 in rat hippocampus attenuated the increase of IL1β expression associated with artificially induced epileptogenesis [135]. FGF1 or FGF2 administration decreased the inflammatory responses associated with acute pancreatitis in mice [136]. Furthermore, FGF1 administration decreased the secretion of TNFα and IL6 in a mouse model of diet-induced obesity [137], and this effect stemmed from the suppression of pro-inflammatory JNK signaling. One can suggest that the effects of FGFs on inflammation could depend on the dose and duration of recombinant FGF application, as well as on the specific tissue context.

### 4.7. Stimulation of Angiogenesis

FGFs were initially discovered as proteins that stimulate the growth of a variety of cell types in vitro. Most striking was their ability to sustain the viability and maintain the proliferation of endothelial cells [138,139], a fundamental observation that made possible the endothelial cell culture. Subsequent in vivo studies (for review see [140,141]) demonstrated that FGF family members, primarily recombinant FGF1 and FGF2, efficiently stimulate angiogenesis, which is the formation of new vessels from preexisting vessels, a process dramatically intensified in the course of trauma repair.

The role of endogenous FGF signaling in repair-related angiogenesis has been demonstrated in a number of studies. For example, the conditional knockout of both FGFR1 and FGFR2 in mouse endothelial cells impaired vascularization during the course of wound healing [142]. Similarly, haploinsufficiency in FGF9 in mice led to a decreased angiogenesis during bone repair, a defect that was rescued by the application of recombinant FGF9 [60]. Neutralizing antibodies against FGF2 suppressed the restorative angiogenesis in a wounded chicken chorioallantoic membrane [143]. In a related context, recombinant FGF2 and FGF1 were demonstrated to efficiently stimulate angiogenesis in rodent models of myocardium ischemia [61], hindlimb ischemia [62,63,64], muscle damage [65], bone repair [66,67], ear ulcer [68], and de-vascularized sternum repair [69].

It is noteworthy that Nagaraja et al. [144], using mathematical modeling based on a compendium of experimental data, identified FGF2 as one of five critical factors required for restorative angiogenesis in wounds with delayed healing. The other four factors include TGFβ, angiopoietin 2, VEGF, and oxygen.

### 4.8. Enhancement of Proteases Expression

The activity of various extracellular proteases is enhanced in the process of trauma repair, resulting in the extracellular matrix (ECM) remodeling and the facilitation of angiogenesis. Zinc-dependent endopeptidases belonging to the group of matrix metalloproteinases (MMP) are especially important for ECM remodeling [145]. A number of studies demonstrated the stimulation of MMP gene expression by FGF1 and FGF2 in various cell types. FGF1 stimulated the expression of MMP1 and MMP3 in endothelial cells [57]. The FGF1-induced increase of the MMP9 expression in malignant mammary epithelial cells was mediated by NFκB signaling [76]. FGF2 stimulated the expression of MMP1 and MMP3 in myofibroblasts [146], and MMP3 [147] and MMP2 [58] in endothelial cells. Transcriptome analysis demonstrated that the treatment of human fibroblasts with FGF2 significantly increased the expression of metalloproteinases MMP1, ADAMTS8, MMP27, MMP10, and MMP3 [74].

Similar to MMPs, plasmin participates in the degradation of ECM [148]. In endothelial cells, recombinant FGF2 increased the expression of the urokinase-type plasminogen activator, a positive regulator of plasmin formation [59]. Apparently, the stimulation of bone repair by FGFs is also related to the enhancement of the protease expression. Indeed, FGF2 enhanced the expression of MMP9 and cathepsin K in osteoclasts [83], thus increasing the bone resorption activity of these cells.

## 5. Tissue Stress and Stimulation of FGF Expression and Release

Canonical FGFs, whose signaling is dependent on HSPGs and FGFRs, function in paracrine or autocrine manners. To efficiently stimulate repair, their local expression and release can be enhanced by various stress factors characteristic of damaged tissues.

### 5.1. FGF Expression

Local hypoxia arising from a circulation impairment is characteristic of traumatic injury. Hypoxia was reported to stimulate the transcription of the *Fgf2* gene in the cultures of cortical neurons [149], radial glia cells in vivo [150], adipose-derived stem cells in vivo [151], endothelial cells [152], and retinal pigment endothelial cells [153]. In macrophages, hypoxia induced the expression of both the *Fgf1* and *Fgf2* genes [154].

Hyperthermia and inflammation in damaged tissues can also be involved in the stimulation of FGF expression. Indeed, heat shock enhanced the FGF1 expression in the cultures of small-airway epithelial cells [155]. The potent proinflammatory cytokine, IL1β, stimulated the *Fgf2* gene transcription in corneal endothelial cells [156], osteoblasts [157], and chondrocytes [158]. Moreover, the IL1β-induced stimulation of the FGF2 expression in corneal endothelial cells was demonstrated to be dependent on NFκB signaling [159]. IL1β also enhanced the expression of the FGF7 (keratinocyte growth factor) in fibroblasts [160].

### 5.2. Release of Signal Peptide-Less FGFs

The majority of secreted canonical FGFs and all hormone-like FGFs possess a cleavable N-terminal signal peptide that is required for their classical secretion through the endoplasmic reticulum (ER) and Golgi apparatus. However, the two most ubiquitously expressed members of the FGF family, FGF1 and FGF2, are devoid of signal peptides and are thus released through nonclassical pathways, independent of ER-Golgi. We have suggested [161] that the loss of signal peptides by FGF1 and FGF2 in the course of evolution enabled a fine regulation of the availability of these proteins, depending on the specific local conditions in the tissue. Indeed, FGF1 export is stimulated by stress conditions, such as heat shock [162], hypoxia [163], and growth factor starvation [164]. Stress-induced FGF1 export requires the formation of a copper-dependent [165] multiprotein complex involving FGF1, S100A13 protein [166], and an alternatively translated 40 kDa form of synaptotagmin 1 [167,168]. The release of FGF1 is also dependent on sphingosine kinase 1 [169], which could serve as a donor of copper ions, as well as a large submembrane protein, AHNAK2 [170], which may function as a platform for the assembly of the FGF1 release complex. It is noteworthy that the stress-induced transmembrane translocation of FGF1 is co-localized with the flipping of the acidic phospholipid phosphatidylserine from the inner to the outer of the cell membrane leaflet [171]. FGF1 secretion is dependent on the existence in the core of the FGF1 of a β-barrel structure [172], which apparently enables the passage of FGF1 through the hydrophobic internal milieu of the plasma membrane phospholipid bilayer.

Trauma is typically accompanied by the severing of cell–cell contacts, which can suppress various signaling pathways relying on these contacts. Thus, Notch signaling depends on the interaction of Notch receptors with their transmembrane ligands, Jagged and Delta, on the surface of neighboring cells. We found that the suppression of Notch signaling induces the release of FGF1 [173,174]. Trauma also leads to the formation of active thrombin from prothrombin. Besides stimulating the formation of fibrin clots, thrombin proteolytically activates PAR receptors. We found that thrombin stimulates the release of FGF1 in a PAR1-dependent manner [175]. Interestingly, an additional stimulating effect of thrombin on FGF1 release is mediated by the proteolytic cleavage of Jagged 1, resulting in the formation of soluble Jagged1, which inhibits Notch signaling [175].

The mechanism of the spontaneous nonclassical export of FGF2 has been extensively studied by the group of Nickel (for a review, see [176]). They found that this process is mediated by the binding of FGF2 to phosphatidylinositol (4.5)-bisphosphate (PI(4.5)P2) localized in the inner leaflet of the cell membrane and the formation of FGF2 oligomers spanning the cell membrane and forming pores. The HSPGs located on the outer surface of the membrane apparently serve as a trap for secreted FGF2. The process of FGF2 secretion was shown to depend on Na,K-ATPase. The direct interaction of FGF2 with the α subunit of Na,K-ATPase is a prerequisite of FGF2 binding to PI(4,5)P2. It remains to be understood how the stress conditions existing in damaged tissues could influence the release of FGF2. Interestingly, we found that the suppression of Notch signaling enhanced FGF2 export (unpublished results). The availability of FGF2 in damaged tissue could be increased by an alternative mechanism: the release from growth factor depots bound to the extracellular matrix (ECM) as a result of the proteolytic degradation of ECM. Thus, elastase activity underlies the release of FGF2 in the cultures of endothelial cells exposed to shear stress [177], and MMP2 stimulated the release of FGF2 from an eye lens capsule [178]. Interestingly, in *Xenopus* embryos, the release of FGFs from ECM by the xHtrA1 protease underlies the long-range FGF signaling in the process of development [179]. In addition to the regulated nonclassical secretion or release from the ECM-bound depots, the increase of the FGF1 and FGF2 bioavailability in trauma could be achieved as a result of necroptotic or pyroptotic cell death, accompanied by major membrane damages. Not only the Lo-FGF2, but also the Hi-FGF2 released from damaged cells could exhibit biological activities that stimulate tissue repair. Indeed, recombinant Hi-FGF2 was shown to activate the canonical FGFR/MAPK signaling pathway [180].

## 6. Conclusions

The members of the FGF family function as potent stimulators of tissue repair. The pro-reparative effects of FGF are based on their abilities to stimulate cell proliferation and migration, enhance angiogenesis, regulate inflammation, maintain cell stemness and promote de-differentiation, protect cells from apoptosis, and stimulate the expression of proteases. The bioavailability of FGFs in damaged tissues is ensured by stress-promoted gene expression and, at least in the case of FGF1, by stress-stimulated release. Many questions remain to be answered in the field of FGF-regulated tissue repair, such as what branches of FGF signaling pathway are responsible for the specific pro-reparative cellular effects of FGFs? What are the relative contributions of the individual cellular effects of FGF to tissue repair? How does the interplay of FGFs with other growth factors and cytokines regulate the repair? What is the role of the negative regulation of FGF signaling in repair processes? What are the mechanisms suppressing the FGF availability in the process of trauma healing? The expanding arsenal of modern methods of genetic analysis has the potential to help in answering these questions.

## Figures and Tables

**Table 1:** Biological effects of FGFs in different cell types.

Cell Type	FGF	Biological Effect	Mechanism (If Known)	Ref.
Endothelial cells	FGF2	Lifespan extension	Telomerase activity increase	[55]
Endothelial cells	FGF2	Apoptosis suppression	Increase of Bcl2 expression	[56]
Endothelial cells	FGF1	Increase of MMP1 and MMP3 expression	Increase of proinflammatory cytokines	[57]
Endothelial cells	FGF2	Increase of MMP2 expression	Activation of FGFR/JNK pathway	[58]
Endothelial cells	FGF2	Induction of uPA expression		[59]
Endothelial cells	FGF1, FGF2, FGF9	Enhancement of angiogenesis	Simulation of proliferation, increase of cell–cell adhesion	[60,61,62,63,64,65,66,67,68,69]
VSMC	FGF1, FGF2	De-differentiation	Suppression of TGFβ signaling	[70,71]
VSMC	FGF1	Increase of proinflammatory cytokines expression		[70]
Fibroblasts	FGF2	Suppression of differentiation to myofibroblasts	Suppression of TGFβ signaling	[72] (review)
Fibroblasts	FGF2	Lifespan extension	Increase of OCT4, SOX2 and NANOG expression	[73]
Fibroblasts	FGF2	Increase of MMP1, ADAMTS8, MMP27, MMP10, and MMP3 expression		[74]
Thyroid Epithelial cells	FGF3	Suppression of differentiation		[75]
Mammary epithelial cells	FGF1	Increase of MMP9 expression	Stimulation of NFκB signaling	[76]
Keratinocytes	FGF2	De-differentiation	Increase of Snail 2 expression	[77]
Keratinocytes	FGF7	Increase of TNFα expression	Activation of FGFR2/Akt/NFκB pathway	[78]
Cardiomyocytes	FGF2	Protection from necrosis	Activation of PI3K/Akt pathway	[79]
Cardiomyocytes	FGF2	Protection from doxorubicin toxic effect	Activation of mTOR/Nrf-2/HO1 pathway	[80]
Chondrocytes	FGF2	De-differentiation		[81]
Chondrocytes	FGF2	Induction of inflammatory phenotype	Mediated by ILβ	[82]
Osteoclasts	FGF2	Increase of MMP9 and cathepsin K expression	Activation of FGFR1/MAPK signaling	[83]
Neurons	FGF1	Suppression of apoptosis	Decrease of the expression of XIAP and caspases 9 and 3	[84]
Neurons	FGF2	Suppression of apoptosis	Activation of PI3K/Akt pathway	[85]
Oligodendrocytes	FGF2	Suppression of apoptosis		[86]
Mesenchymal stem cells	FGF2	Suppression of apoptosis	Increase of Bcl2 expression	[87]
Mesenchymal stem cells	FGF2	Suppression of senescence	Decrease of p16, p21 and p53 expression	[88]
Male germ cells	FGF4	Suppression of apoptosis		[89]

## Abbreviations

ECM	extracellular matrix
FGF	fibroblast growth factor
FGFR	FGF receptor
HSPG	heparan sulfate proteoglycan
MMP	matrix metalloproteinase
VSMC	vascular smooth muscle cell
